# Dibromido(di-2-pyridyl sulfide-κ^2^ *N,N*′)zinc(II)

**DOI:** 10.1107/S1600536808000081

**Published:** 2008-01-09

**Authors:** Mario Wriedt, Inke Jess, Christian Näther

**Affiliations:** a Institut für Anorganische Chemie, Christian-Albrechts-Universität Kiel, Olshausenstrasse 40, D-24098 Kiel, Germany

## Abstract

The molecule of the title compound, [ZnBr_2_(C_10_H_8_N_2_S)], contains a six-membered chelate ring in a boat conformation in which the Zn atom is coordinated by two Br atoms and by the two pyridyl N atoms of a single di-2-pyridyl sulfide (dps) ligand within a slightly distorted tetra­hedron. The dihedral angle between the pyridine rings is 52.7 (1)°. As is usual for this type of complex, the sulfide group does not participate in the zinc coordination.

## Related literature

For related literature, see: Anderson & Steel (1998 ▶); Bhosekar *et al.* (2007 ▶); Kondo *et al.* (1995 ▶); Nicolò *et al.* (1996 ▶); Teles *et al.* (1999 ▶); Tresoldi *et al.* (1991 ▶, 1992 ▶); Näther *et al.* (2003 ▶); Näther & Jess (2006 ▶)
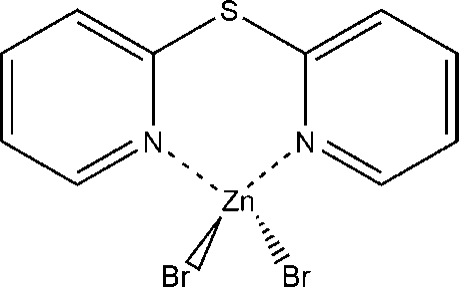


## Experimental

### 

#### Crystal data

[ZnBr_2_(C_10_H_8_N_2_S)]
*M* *_r_* = 413.43
Monoclinic, 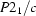

*a* = 11.0385 (8) Å
*b* = 8.9627 (5) Å
*c* = 13.157 (1) Å
β = 91.663 (9)°
*V* = 1301.2 (2) Å^3^
*Z* = 4
Mo *K*α radiation
μ = 8.16 mm^−1^
*T* = 170 (2) K
0.14 × 0.10 × 0.07 mm

#### Data collection

Stoe IPDS-1 diffractometer
Absorption correction: numerical (*X-SHAPE*; Stoe, 1998 ▶) *T*_min_ = 0.285, *T*_max_ = 0.394
14781 measured reflections
3105 independent reflections
2534 reflections with *I* > 2σ(*I*)
*R*_int_ = 0.041

#### Refinement

*R*[*F* ^2^ > 2σ(*F* ^2^)] = 0.033
*wR*(*F* ^2^) = 0.085
*S* = 1.03
3105 reflections
146 parameters
H-atom parameters constrained
Δρ_max_ = 1.15 e Å^−3^
Δρ_min_ = −1.16 e Å^−3^

### 

Data collection: *IPDS Program Package* (Stoe, 1998 ▶); cell refinement: *IPDS Program Package*; data reduction: *IPDS Program Package*; program(s) used to solve structure: *SHELXS97* (Sheldrick, 2008 ▶); program(s) used to refine structure: *SHELXL97* (Sheldrick, 2008 ▶); molecular graphics: *XP* in *SHELXTL* (Bruker, 1998 ▶); software used to prepare material for publication: *CIFTAB* in *SHELXTL*.

## Supplementary Material

Crystal structure: contains datablocks I, global. DOI: 10.1107/S1600536808000081/im2053sup1.cif

Structure factors: contains datablocks I. DOI: 10.1107/S1600536808000081/im2053Isup2.hkl

Additional supplementary materials:  crystallographic information; 3D view; checkCIF report

## Figures and Tables

**Table:** 

Zn1—N11	2.055 (3)
Zn1—N1	2.058 (3)
Zn1—Br2	2.3504 (6)
Zn1—Br1	2.3527 (5)

**Table:** 

N11—Zn1—N1	94.66 (11)
N11—Zn1—Br2	115.35 (8)
N1—Zn1—Br2	109.76 (8)
N11—Zn1—Br1	107.43 (8)
N1—Zn1—Br1	108.56 (8)
Br2—Zn1—Br1	118.38 (2)

**Fig. 1.**
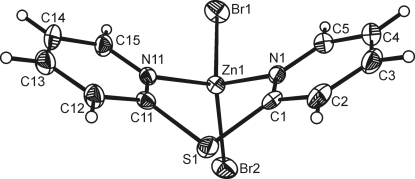
: Crystal structure of compound I showing the labelling scheme, and displacement ellipsoids drawn at the 50% probability level.

## supplementary crystallographic information

### Comment

In our ongoing investigation on the synthesis, structures and properties of new coordination polymers based on zinc(II) halides and N-donor ligands (Bhosekar *et al. *2007), we have started systematic investigation of their thermal behavior because we have demonstrated that new ligand-deficient coordination polymers can be conveniently prepared by thermal decomposition of suitable ligand-rich precursur compounds (Näther *et al.* 2003; Näther & Je\&s, 2006). In further investigations we have reacted zinc(II) bromide with 2,2'-bipyridyldisulfide (dpds). In this reaction a cleavage of the S—S bond takes place leading to the formation of di-2-pyridyl sulfide (dps) which in a concomitant reaction with zinc(II) bromide forms the title chelate-complex.

In general, dps is a versatile ambidentate ligand that, due to its conformational flexibility, can act in N,*N*'-bidentate (Tresoldi *et al.*, 1992; Kondo *et al.*, 1995 and Nicolò *et al.*, 1996) or bridging (Tresoldi *et al.*, 1991 and Teles *et al.*, 1999) coordination modes toward many metals, resulting in complexes with different stereochemistry. When dps is connected to a metal atom as a chelate ligand, a six-membered ring in boat conformation is formed, differently from its rigid analogues 2,2'-bipyridine that generates a pentacyclic chelate in a planar arragement. In addition, in some cases dps can act as tridentate ligand in a N,*N*,*S*-coordination mode involving metal-sulfur interactions (Anderson & Steel, 1998).

In the molecular structure the coordination geometry about the Zn atom is almost tetrahedral with bonds being formed to two Br atoms and the two pyridyl N atoms of a single dps ligand (Fig. 1). These interactions result in the formation of a six-membered chelate ring in a boat conformation. The *X*—Zn—*X* angles (*X* = Br, N) range from 94.7 (1) to 118.38 (2)°, the largest being Br—Zn—Br. The Zn—Br and Zn—N distances are in the range of 2.3504 (6)–2.3527 (5) and 2.055 (3)–2.058 (3) Å. The structural parameters in the bps molecule are quite regular. In particular the C—S bonds of 1.776 (4) Å (S1—C1) and 1.778 (4) Å (S1—C11) are in good agreement with those expected for C(*sp*^2^)—S bonds (1.77 Å, Tresoldi *et al.* 1992).

### Experimental

ZnBr_2_ and 2,2'-bipyridyldisulfide was obtained from Alfa Aesar, methanol was obtained from Fluka. 0.125 mmol (28.1 mg) zinc(II) bromide, 0.0312 mmol (6.87 mg) 2,2'-bipyridyldisulfide and 3 ml of methanol were transfered into a test-tube, which was closed and heated to 110 °C for three days. On cooling colourless block-shaped single crystals of the title compound were obtained.

### Refinement

All H atoms were located from the difference Fourier map but were positioned with idealized geometry and were refined isotropically with *U*_eq_(H) = 1.2 *U*_eq_(C) of the parent atom using a riding model with C—H = 0.97 Å.

### Crystal data

**Table:**

[ZnBr_2_(C_10_H_8_N_2_S)]	*F*_000_ = 792
*M**_r_* = 413.43	*D*_x_ = 2.111 Mg m^−^^3^
Monoclinic, *P*2_1_/*c*	Mo *K*α radiation λ = 0.71073 Å
Hall symbol: -P 2ybc	Cell parameters from 8000 reflections
*a* = 11.0385 (8) Å	θ = 13.8–24.9º
*b* = 8.9627 (5) Å	µ = 8.16 mm^−^^1^
*c* = 13.157 (1) Å	*T* = 170 (2) K
β = 91.663 (9)º	Block, colourless
*V* = 1301.2 (2) Å^3^	0.14 × 0.10 × 0.07 mm
*Z* = 4	

### Data collection

**Table:**

Stoe IPDS-1 diffractometer	3105 independent reflections
Radiation source: fine-focus sealed tube	2534 reflections with *I* > 2σ(*I*)
Monochromator: graphite	*R*_int_ = 0.041
*T* = 170(2) K	θ_max_ = 28.0º
Phi scans	θ_min_ = 2.8º
Absorption correction: numerical(X-SHAPE; Stoe, 1998)	*h* = −14→14
*T*_min_ = 0.285, *T*_max_ = 0.394	*k* = −11→11
14781 measured reflections	*l* = −17→17

### Refinement

**Table:**

Refinement on *F*^2^	Hydrogen site location: inferred from neighbouring sites
Least-squares matrix: full	H-atom parameters constrained
*R*[*F*^2^ > 2σ(*F*^2^)] = 0.033	*w* = 1/[σ^2^(*F*_o_^2^) + (0.0528*P*)^2^ + 0.9116*P*] where *P* = (*F*_o_^2^ + 2*F*_c_^2^)/3
*wR*(*F*^2^) = 0.085	(Δ/σ)_max_ = 0.001
*S* = 1.03	Δρ_max_ = 1.15 e Å^−^^3^
3105 reflections	Δρ_min_ = −1.15 e Å^−^^3^
146 parameters	Extinction correction: SHELXL, Fc^*^=kFc[1+0.001xFc^2^λ^3^/sin(2θ)]^-1/4^
Primary atom site location: structure-invariant direct methods	Extinction coefficient: 0.0076 (6)
Secondary atom site location: difference Fourier map	

### Special details

**Table:**

Geometry. All e.s.d.'s (except the e.s.d. in the dihedral angle between two l.s. planes) are estimated using the full covariance matrix. The cell e.s.d.'s are taken into account individually in the estimation of e.s.d.'s in distances, angles and torsion angles; correlations between e.s.d.'s in cell parameters are only used when they are defined by crystal symmetry. An approximate (isotropic) treatment of cell e.s.d.'s is used for estimating e.s.d.'s involving l.s. planes.
Refinement. Refinement of *F*^2^ against ALL reflections. The weighted *R*-factor *wR* and goodness of fit *S* are based on *F*^2^, conventional *R*-factors *R* are based on *F*, with *F* set to zero for negative *F*^2^. The threshold expression of *F*^2^ > σ(*F*^2^) is used only for calculating *R*-factors(gt) *etc*. and is not relevant to the choice of reflections for refinement. *R*-factors based on *F*^2^ are statistically about twice as large as those based on *F*, and *R*- factors based on ALL data will be even larger.

### Fractional atomic coordinates and isotropic or equivalent isotropic displacement parameters (Å^2^)

**Table:**

	*x*	*y*	*z*	*U*_iso_*/*U*_eq_	
Zn1	0.74601 (4)	0.84740 (4)	0.50911 (3)	0.01791 (12)	
Br1	0.72001 (4)	1.08853 (4)	0.57571 (3)	0.03251 (13)	
Br2	0.76278 (4)	0.82087 (5)	0.33235 (3)	0.03579 (13)	
N1	0.8929 (3)	0.7499 (3)	0.5825 (2)	0.0174 (5)	
C1	0.8968 (3)	0.6003 (4)	0.5939 (2)	0.0187 (6)	
C2	0.9955 (4)	0.5287 (4)	0.6405 (3)	0.0258 (7)	
H2	0.9973	0.4231	0.6461	0.031*	
C3	1.0912 (4)	0.6142 (5)	0.6789 (3)	0.0308 (8)	
H3	1.1597	0.5677	0.7106	0.037*	
C4	1.0854 (4)	0.7682 (5)	0.6702 (3)	0.0323 (9)	
H4	1.1491	0.8289	0.6973	0.039*	
C5	0.9853 (3)	0.8322 (4)	0.6215 (3)	0.0252 (7)	
H5	0.9818	0.9377	0.6153	0.030*	
S1	0.77889 (9)	0.48727 (9)	0.54086 (8)	0.0262 (2)	
N11	0.6206 (3)	0.7109 (3)	0.5746 (2)	0.0173 (5)	
C11	0.6435 (3)	0.5653 (3)	0.5880 (2)	0.0175 (6)	
C12	0.5613 (3)	0.4683 (4)	0.6314 (3)	0.0247 (7)	
H12	0.5790	0.3650	0.6384	0.030*	
C13	0.4528 (4)	0.5268 (4)	0.6640 (3)	0.0291 (8)	
H13	0.3945	0.4635	0.6935	0.035*	
C14	0.4303 (4)	0.6768 (5)	0.6535 (3)	0.0311 (8)	
H14	0.3569	0.7185	0.6767	0.037*	
C15	0.5160 (3)	0.7667 (4)	0.6085 (3)	0.0240 (7)	
H15	0.5004	0.8705	0.6015	0.029*	

### Atomic displacement parameters (Å^2^)

**Table:**

	*U*^11^	*U*^22^	*U*^33^	*U*^12^	*U*^13^	*U*^23^
Zn1	0.0211 (2)	0.01532 (19)	0.01722 (19)	−0.00099 (14)	−0.00055 (14)	0.00326 (13)
Br1	0.0418 (3)	0.01468 (17)	0.0407 (2)	0.00437 (14)	−0.00482 (17)	−0.00135 (13)
Br2	0.0492 (3)	0.0418 (2)	0.01642 (18)	−0.01221 (18)	0.00179 (15)	0.00378 (14)
N1	0.0158 (14)	0.0174 (12)	0.0192 (13)	0.0002 (10)	0.0023 (10)	−0.0007 (10)
C1	0.0196 (17)	0.0198 (15)	0.0171 (15)	0.0046 (12)	0.0062 (12)	−0.0003 (11)
C2	0.026 (2)	0.0284 (17)	0.0238 (17)	0.0106 (14)	0.0068 (14)	0.0047 (13)
C3	0.0200 (19)	0.049 (2)	0.0232 (17)	0.0120 (16)	0.0013 (14)	0.0013 (15)
C4	0.0188 (19)	0.047 (2)	0.0313 (19)	0.0016 (16)	−0.0014 (15)	−0.0104 (17)
C5	0.0214 (19)	0.0282 (17)	0.0261 (18)	−0.0005 (14)	0.0007 (14)	−0.0044 (14)
S1	0.0246 (5)	0.0165 (4)	0.0380 (5)	−0.0023 (3)	0.0090 (4)	−0.0081 (3)
N11	0.0172 (14)	0.0191 (12)	0.0157 (12)	−0.0031 (10)	−0.0003 (10)	0.0008 (10)
C11	0.0208 (18)	0.0151 (13)	0.0164 (14)	−0.0039 (12)	−0.0003 (12)	−0.0008 (11)
C12	0.0234 (19)	0.0238 (16)	0.0270 (18)	−0.0072 (14)	0.0021 (14)	0.0013 (13)
C13	0.027 (2)	0.0340 (19)	0.0269 (18)	−0.0118 (16)	0.0030 (15)	0.0007 (14)
C14	0.0142 (18)	0.041 (2)	0.038 (2)	−0.0009 (15)	0.0047 (15)	−0.0051 (16)
C15	0.0164 (18)	0.0251 (16)	0.0305 (18)	0.0017 (13)	0.0014 (13)	−0.0014 (13)

### Geometric parameters (Å, °)

**Table:**

Zn1—N11	2.055 (3)	C4—H4	0.9500
Zn1—N1	2.058 (3)	C5—H5	0.9500
Zn1—Br2	2.3504 (6)	S1—C11	1.778 (4)
Zn1—Br1	2.3527 (5)	N11—C11	1.340 (4)
N1—C5	1.348 (4)	N11—C15	1.347 (5)
N1—C1	1.350 (4)	C11—C12	1.391 (5)
C1—C2	1.391 (5)	C12—C13	1.387 (6)
C1—S1	1.776 (4)	C12—H12	0.9500
C2—C3	1.388 (6)	C13—C14	1.373 (6)
C2—H2	0.9500	C13—H13	0.9500
C3—C4	1.386 (6)	C14—C15	1.388 (5)
C3—H3	0.9500	C14—H14	0.9500
C4—C5	1.385 (5)	C15—H15	0.9500
			
N11—Zn1—N1	94.66 (11)	N1—C5—H5	118.9
N11—Zn1—Br2	115.35 (8)	C4—C5—H5	118.9
N1—Zn1—Br2	109.76 (8)	C1—S1—C11	104.63 (15)
N11—Zn1—Br1	107.43 (8)	C11—N11—C15	118.6 (3)
N1—Zn1—Br1	108.56 (8)	C11—N11—Zn1	120.5 (2)
Br2—Zn1—Br1	118.38 (2)	C15—N11—Zn1	120.8 (2)
C5—N1—C1	118.6 (3)	N11—C11—C12	122.7 (3)
C5—N1—Zn1	121.6 (2)	N11—C11—S1	119.7 (2)
C1—N1—Zn1	119.8 (2)	C12—C11—S1	117.5 (3)
N1—C1—C2	122.0 (3)	C13—C12—C11	118.0 (3)
N1—C1—S1	120.1 (3)	C13—C12—H12	121.0
C2—C1—S1	117.8 (3)	C11—C12—H12	121.0
C3—C2—C1	118.9 (3)	C14—C13—C12	119.7 (3)
C3—C2—H2	120.5	C14—C13—H13	120.2
C1—C2—H2	120.5	C12—C13—H13	120.2
C4—C3—C2	119.2 (3)	C13—C14—C15	119.1 (4)
C4—C3—H3	120.4	C13—C14—H14	120.4
C2—C3—H3	120.4	C15—C14—H14	120.4
C5—C4—C3	119.0 (4)	N11—C15—C14	121.8 (3)
C5—C4—H4	120.5	N11—C15—H15	119.1
C3—C4—H4	120.5	C14—C15—H15	119.1
N1—C5—C4	122.3 (3)		
